# Nosocomial SARS-CoV-2 Infections and Mortality During Unique COVID-19 Epidemic Waves

**DOI:** 10.1001/jamanetworkopen.2023.41936

**Published:** 2023-11-10

**Authors:** Nishi Dave, Daniel Sjöholm, Pontus Hedberg, Anders Ternhag, Fredrik Granath, Janneke D. M. Verberk, Anders F. Johansson, Suzanne D. van der Werff, Pontus Nauclér

**Affiliations:** 1 Department of Medicine, Solna, Division of Infectious Diseases, Karolinska Institutet, Stockholm, Sweden; 2 Department of Medicine, Clinical Epidemiology Division, Karolinska Institutet, Stockholm, Sweden; 3 Department of Infectious Diseases, Karolinska University Hospital, Stockholm, Sweden; 4 Department of Medical Microbiology and Infection Prevention, University Medical Centre Utrecht, Utrecht, the Netherlands; 5 Department of Epidemiology, Julius Centre for Health Sciences and Primary Care, University Medical Centre Utrecht, Utrecht, the Netherlands; 6 Department of Clinical Microbiology and Laboratory for Molecular Infection Medicine Sweden, Umeå University, Umeå, Sweden

## Abstract

**Importance:**

Quantifying the burden of nosocomial SARS-CoV-2 infections and associated mortality is necessary to assess the need for infection prevention and control measures.

**Objective:**

To investigate the occurrence of nosocomial SARS-CoV-2 infections and associated 30-day mortality among patients admitted to hospitals in Region Stockholm, Sweden.

**Design, Setting, and Participants:**

A retrospective, matched cohort study divided the period from March 1, 2020, until September 15, 2022, into a prevaccination period, early vaccination and pre-Omicron (period 1), and late vaccination and Omicron (period 2). From among 303 898 patients 18 years or older living in Region Stockholm, 538 951 hospital admissions across all hospitals were included. Hospitalized admissions with nosocomial SARS-CoV-2 infections were matched to as many as 5 hospitalized admissions without nosocomial SARS-CoV-2 by age, sex, length of stay, admission time, and hospital unit.

**Exposure:**

Nosocomial SARS-CoV-2 infection defined as the first positive polymerase chain reaction test result at least 8 days after hospital admission or within 2 days after discharge.

**Main Outcomes and Measures:**

Primary outcome of 30-day mortality was analyzed using time-to-event analyses with a Cox proportional hazards regression model adjusted for age, sex, educational level, and comorbidities.

**Results:**

Among 2193 patients with SARS-CoV-2 infections or reinfections (1107 women [50.5%]; median age, 80 [IQR, 71-87] years), 2203 nosocomial SARS-CoV-2 infections were identified. The incidence rate of nosocomial SARS-CoV-2 infections was 1.57 (95% CI, 1.51-1.64) per 1000 patient-days. In the matched cohort, 1487 hospital admissions with nosocomial SARS-CoV-2 infections were matched to 5044 hospital admissions without nosocomial SARS-CoV-2 infections. Thirty-day mortality was higher in the prevaccination period (adjusted hazard ratio [AHR], 2.97 [95% CI, 2.50-3.53]) compared with period 1 (AHR, 2.08 [95% CI, 1.50-2.88]) or period 2 (AHR, 1.22 [95% CI, 0.92-1.60]). Among patients with nosocomial SARS-CoV-2 infections, 30-day AHR comparing those with 2 or more doses of SARS-CoV-2 vaccination and those with less than 2 doses was 0.64 (95% CI, 0.46-0.88).

**Conclusions and Relevance:**

In this matched cohort study, nosocomial SARS-CoV-2 infections were associated with higher 30-day mortality during the early phases of the pandemic and lower mortality during the Omicron variant wave and after the introduction of vaccinations. Mitigation of excess mortality risk from nosocomial transmission should be a strong focus when population immunity is low through implementation of adequate infection prevention and control measures.

## Key Points

**Question:**

What is the incidence of nosocomial SARS-CoV-2 infections and associated 30-day mortality among patients admitted to hospitals in Region Stockholm, Sweden?

**Findings:**

In this cohort study, 2203 hospital admissions in 2193 patients were identified with nosocomial SARS-CoV-2 infection between March 1, 2020, and September 15, 2022. Thirty-day mortality was higher during the early phases of the pandemic; mortality was lower during the Omicron variant wave and after introduction of vaccinations.

**Meaning:**

These findings suggest that infection prevention and control measures mitigating excess mortality risk from nosocomial transmission are important when population immunity is low; the role of these measures to prevent excess deaths is reduced with successful vaccination and/or less severe virus variants.

## Teaser

This cohort study describes the incidence rate of nosocomial SARS-CoV-2 infections across all hospitals in Region Stockholm, Sweden, during the COVID-19 pandemic and investigates associated 30-day mortality.

## Introduction

Hospitalized patients are at risk of SARS-CoV-2 acquisition, with nosocomial SARS-CoV-2 infections reported to have higher mortality risk.^1^ A systematic review of studies from 2020 to the beginning of 2021 reported a 1.3 times greater risk of mortality among individuals with nosocomial vs community-acquired SARS-CoV-2 infections.^2^ Most studies^3,4^ compared individuals hospitalized with community-acquired SARS-CoV-2 infections and individuals with nosocomial infections, precluding investigation of the excess mortality due to SARS-CoV-2 infections among hospitalized patients. Additionally, studies analyzing nosocomial SARS-CoV-2 infections and the association with severe outcomes after the emergence of Omicron variants are scarce.^3,4,5^

To account for underlying changes over time in SARS-CoV-2 incidence, prevalence, and changing population immunity to the virus, outcomes of nosocomial SARS-CoV-2 infections necessitate analyses among patients with similar characteristics and across different time periods during the pandemic. Such analyses, including risk quantification, may guide prevention efforts, including planning and implementation of infection prevention and control (IPC) measures. In this study, the primary aim was to describe the incidence rate of nosocomial SARS-CoV-2 infections across all hospitals in Region Stockholm, Sweden, between March 1, 2020, and September 15, 2022, and to investigate the associated 30-day mortality. The secondary aim was to assess the 31- to 90-day mortality, number of hospital-free days (HFD) with a follow-up of 30 days among patients with and without nosocomial SARS-CoV-2 infections, and the association between vaccination and mortality given a SARS-CoV-2 infection.

## Methods

### Population and Data Sources

We conducted a retrospective, population-based observational cohort study including all individuals 18 years or older residing in Region Stockholm for at least 3 years prior to the study start on March 1, 2020 (N = 1 723 985), to adequately capture underlying health care conditions. From this population, we identified all individuals (n = 303 898) with hospital admissions until September 15, 2022. The study was approved by the Swedish Ethical Review Board, which waived the need for informed consent since analyses were based on retrospectively collected data from the administrative health registry. This study followed the Strengthening the Reporting of Observational Studies in Epidemiology (STROBE) reporting guideline.

Information on hospital admissions, patient demographic characteristics, population data, comorbidities, hospital unit, and date of death was obtained from the Region Stockholm Healthcare Data Warehouse database^6^; educational level, from the Longitudinal Integrated Database for Health Insurance and Labour Market Studies database, Statistics Sweden^7^; and vaccination data from the national vaccination register at the Public Health Agency of Sweden.^8^ Polymerase chain reaction (PCR) test results positive for SARS-CoV-2 were obtained from SmiNet, the register of notifiable diseases.^9^ SARS-CoV-2 PCR information was complemented with negative test results, sequencing, and cycle thresholds values from the Quality Register for SARS-CoV-2 (COVID-19),^10^ which collects data from laboratory systems, and the Intelligence (TakeCare)^11^ database, which contains electronic health record case notes. Data were linked using personal identification numbers that all Swedish residents have.

### Variable Definitions

To account for the epidemic and vaccination variation, 3 time periods were defined. The prevaccination period was from March 1, 2020, until January 24, 2021. The end date was defined as 4 weeks after the first public campaign of SARS-CoV-2 vaccination in Region Stockholm.^12^ Two postvaccination periods were defined using sequencing data from SmiNet: period 1 from January 25, 2021, until January 6, 2022, and period 2 from January 7 until September 15, 2022, in which the Omicron variant accounted for at least 90% of all sampled sequences in Region Stockholm. Hospital admissions with a positive SARS-CoV-2 PCR test result were defined as nosocomial if the test was performed on day 8 or later after admission or up to 2 days after discharge, given a length of stay (LOS) of at least 8 days; as community-acquired if the test was performed 14 days before or up to 2 days after admission; and as indeterminate if the test was performed between 3 to 7 days after admission.^13^ Comorbidities, based on codes from the *International Statistical Classification of Diseases and Related Health Problems, Tenth Revision*, were grouped as cancer, cardiovascular diseases, chronic kidney diseases, chronic lung diseases, diabetes, hypertension, and immunosuppression (eTable 1 in Supplement 1).

### Study Design and Outcomes

The proportion of community-acquired, indeterminate, and nosocomial SARS-CoV-2 infections along with incidence rate of nosocomial SARS-CoV-2 was estimated using data on all SARS-CoV-2 infections (including reinfections). Reinfection was defined as obtaining a positive SARS-CoV-2 PCR test result more than 90 days after a previous positive test result.

Analyses between nosocomial infection and outcomes were restricted to the first positive SARS-CoV-2 infection for each patient. Excluding community-acquired and indeterminate SARS-CoV-2 infections, a cohort was created by matching patient admissions with nosocomial SARS-CoV-2 infection, hereafter referred to as COVID-19 admissions, with patient admissions without SARS-CoV-2 infection, hereafter referred to as non–COVID-19 admissions. Using sampling with replacement^14^ and a nearest matching method, up to 5 non–COVID-19 admissions were matched to each COVID-19 admission on the following matching variables: age (±2 years), sex (exact match), calendar time of admission (non–COVID-19 admission date within ±30 days of matched COVID-19 admission date), hospital unit where the SARS-CoV-2 positive PCR test result was obtained (patient with a non–COVID-19 admission was in the same hospital unit as the patient with a COVID-19 admission), and days from admission until index date. The index date for COVID-19 admissions was date of the positive SARS-CoV-2 test result and for non–COVID-19 admissions, the date of the non–COVID-19 admission plus days it took for the matched patient with COVID-19 to have a positive SARS-CoV-2 test result. Thus, all matched admissions had the same LOS up until the index date (eMethods in Supplement 1).

The primary outcome was 30-day mortality from the index date. Secondary outcomes were 31- to 90-day mortality and HFD (days alive and out of hospital) within the first 30 days after the index date.

### Statistical Analysis

The incidence rate of nosocomial SARS-CoV-2 was assessed using a 14-day moving mean. Calculation of patient-days at risk used admissions with LOS greater than 7 days. All first positive PCR test results among community SARS-CoV-2 infections were plotted to compare the infection burden in the community. Categorical data were presented as frequencies and percentages; continuous data, as medians and IQR.

Kaplan-Meier curves were plotted and compared using a log-rank test to assess mortality in groups with COVID-19 and non–COVID-19 admissions. Cox proportional hazards regression models were used to investigate the association with mortality, adjusted for age, sex, educational level, and comorbidities. Age was modeled using restricted cubic splines to account for nonlinear effects. The 31- to 90-day mortality was analyzed by restricting COVID-19 admissions and their matched non–COVID-19 admissions to March 1, 2020, until July 15, 2022, to allow for a 90-day follow-up period. The median and 95% CIs of HFD was assessed using the bootstrap method and boot package in R, version 4.1.0 (R Project for Statistical Computing).^15^ Two sensitivity analyses were performed. First, we restricted the matched cohort to patients with cycle threshold values of less than 30 or those with sequenced PCR samples and their matched controls in period 2, to account for residual viral debris from prior acute infections. Second, we changed the cutoff for nosocomial infection from 8 to 5 days to account for shorter incubation period of the Omicron variant.

The association between vaccination and 30-day mortality was investigated among patient admissions with nosocomial SARS-CoV-2 in periods 1 and 2, comparing individuals with at least a primary course (≥2 vaccine doses) with patients who had less than 2 vaccine doses using Cox proportional hazards regression models as described above. Individuals were considered as vaccinated 14 days after receiving the vaccine. Two-sided *P* < .05 indicated statistical significance.

## Results

### Occurrence of Nosocomial SARS-CoV-2 Infections

**Figure 1.**
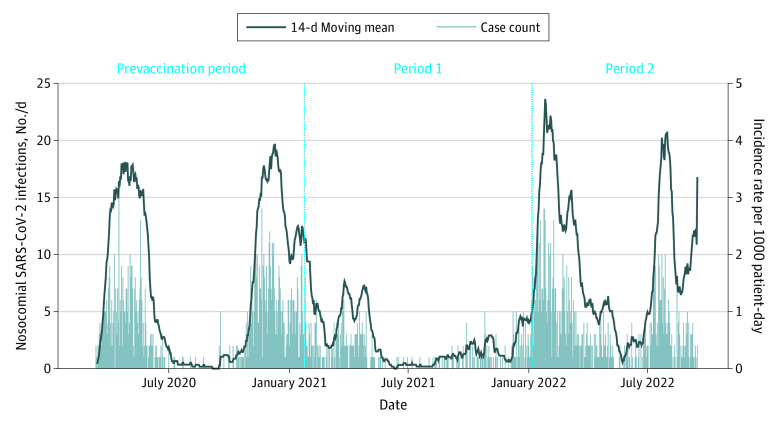
Incidence Rate per 1000 Patient-Days and Daily Count of Nosocomial SARS-CoV-2 Infections The left axis shows the daily count of nosocomial SARS-CoV-2 infections between March 2020 and September 2022. The right axis shows the incidence rate of nosocomial SARS-CoV-2 infections between March 2020 and September 2022 calculated using a 14-day moving mean. To calculate the patient-days at risk, only admissions with length of stay greater than 7 days were included.

From a total of 438 640 SARS-CoV-2 infections occurring between March 1, 2020, until September 15, 2022, in Region Stockholm, 2203 nosocomial SARS-CoV-2 infections among 2193 patients were identified. Patients had a median age of 80 (IQR, 71-87) years; 1107 (50.5%) were women, 1086 (49.5%) were men, and 970 (44.2%) had a secondary educational level. The overall incidence rate of nosocomial SARS-CoV-2 was 1.57 (95% CI, 1.51-1.64) per 1000 patient-days, with peaks following high transmission in the community (Figure 1 and eFigure 1 in Supplement 1); however, extensive community testing was not available until after June 2020 nor after February 2022; before June 2020 and after February 2022, community testing was only available to certain population groups such as the vulnerable, and there were no mass public testing practices. The incidence rate was 1.79 (95% CI, 1.68-1.90) in the prevaccination period, 0.94 (95% CI, 0.86-1.03) in period 1, and 2.25 (95% CI, 2.09-2.43) in period 2.

Of 28 909 hospital admissions with any SARS-CoV-2 infection, 26 121 were community-acquired (90.4%), 585 (2.0%) were indeterminate, and 2203 (7.6%) were nosocomial. Of nosocomial infections, 1036 of 13 481 (7.7%) were nosocomial in the prevaccination period, 486 of 8037 (6.0%) in period 1, and 681 of 7391 (9.2%) in period 2 (eFigure 2 in Supplement 1).

### Outcomes Among COVID-19 Compared With Non–COVID-19 Admissions

Among 2203 COVID-19 admissions, 2120 occurred in patients with a first positive nosocomial SARS-CoV-2 infection. Median time from admission to positive SARS-CoV-2 test result was 14 (IQR, 10-23) days in the prevaccination period, 17 (IQR, 12-30) days in period 1, and 13 (IQR, 10-18) days in period 2. We matched 1487 COVID-19 admissions with 5044 non–COVID-19 admissions, which yielded 2 groups with similar demographic characteristics and comorbidities (Table 1). Also, the proportion of negative test results at admission, diagnosis at admission via the emergency department, and in-patient diagnosis between admission and index date were similar between the 2 groups (eTable 2 and eFigure 3 in Supplement 1). The median age was 81 (IQR, 75-87) years in the COVID-19 group, 783 (52.7%) were women and 704 (47.3%) were men, and 947 (63.7%) had hypertension (Table 1). COVID-19 admissions not included in the matched cohort had similar demographic characteristics in terms of educational level and comorbidities; however, they were younger (median age, 74 [IQR, 60-84] years) and had a higher proportion of men (344 of 633 [54.3%]) (eTable 3 in Supplement 1).

**Table 1.** Demographic Characteristics and Outcomes Among COVID-19 vs Non–COVID-19 Groups^a^

Characteristic	All COVID-19 cases (n = 2120)^b^	Matched cohort by study group							
		Whole study period		Prevaccination period		Period 1		Period 2	
		COVID-19 (n = 1487)	Non–COVID-19 (n = 5044)	COVID-19 (n = 705)	Non–COVID-19 (n = 2427)	COVID-19 (n = 286)	Non–COVID-19 (n = 876)	COVID-19 (n = 496)	Non–COVID-19 (n = 1741)
Sex									
Women	1072 (50.6)	783 (52.7)	2753 (54.6)	370 (52.5)	1305 (53.8)	158 (55.2)	491 (56.1)	255 (51.4)	957 (55.0)
Men	1048 (49.4)	704 (47.3)	2291 (45.4)	335 (47.5)	1122 (46.2)	128 (44.8)	385 (43.9)	241 (48.6)	784 (45.0)
Age, median (IQR), y	80 (71-87)	81 (75-87)	83 (77-88)	82 (75-88)	83 (77-89)	79 (72-86)	81 (75-87)	82 (75-88)	83 (77-89)
No. of vaccine doses at admission date									
0	1258 (59.3)	856 (57.6)	2834 (56.2)	705 (100)	2425 (99.9)	107 (37.4)	302 (34.5)	44 (8.9)	107 (6.1)
1	64 (3.0)	46 (3.1)	104 (2.1)	0	2 (0.1)	35 (12.2)	84 (9.6)	11 (2.2)	18 (1.0)
2	263 (12.4)	164 (11.0)	478 (9.5)	0	0	96 (33.6)	324 (37.0)	68 (13.7)	154 (8.8)
3	364 (17.2)	287 (19.3)	1044 (20.7)	0	0	48 (16.8)	166 (18.9)	239 (48.2)	878 (50.4)
4	169 (8.0)	132 (8.9)	581 (11.5)	0	0	0	0	132 (26.6)	581 (33.4)
5	2 (0.1)	2 (0.1)	3 (0.1)	0	0	0	0	2 (0.4)	3 (0.2)
Comorbidities^c^									
Cancer	462 (21.8)	322 (21.7)	1071 (21.2)	170 (24.1)	555 (22.9)	52 (18.2)	171 (19.5)	100 (20.2)	345 (19.8)
Cardiovascular diseases	946 (44.6)	697 (46.9)	2355 (46.7)	363 (51.5)	1231 (50.7)	134 (46.9)	358 (40.9)	200 (40.3)	766 (44.0)
Chronic kidney diseases	337 (15.9)	244 (16.4)	785 (15.6)	143 (20.3)	434 (17.9)	35 (12.2)	125 (14.3)	66 (13.3)	226 (13.0)
Chronic lung diseases	392 (18.5)	288 (19.4)	890 (17.6)	164 (23.3)	448 (18.5)	46 (16.1)	139 (15.9)	78 (15.7)	303 (17.4)
Diabetes	512 (24.2)	368 (24.7)	1231 (24.4)	188 (26.7)	603 (24.8)	60 (21.0)	195 (22.3)	120 (24.2)	433 (24.9)
Hypertension	1289 (60.8)	947 (63.7)	3358 (66.6)	453 (64.3)	1657 (68.3)	183 (64.0)	570 (65.1)	311 (62.7)	1131 (65.0)
Immunosuppression	130 (6.1)	86 (5.8)	213 (4.2)	55 (7.8)	132 (5.4)	13 (4.5)	32 (3.7)	18 (3.6)	49 (2.8)
Educational level									
Primary	567 (26.7)	413 (27.8)	1415 (28.1)	202 (28.7)	696 (28.7)	80 (28.0)	271 (30.9)	131 (26.4)	448 (25.7)
Secondary	932 (44.0)	628 (42.2)	2075 (41.1)	281 (40.0)	973 (40.1)	131 (45.8)	345 (39.4)	216 (43.5)	757 (43.5)
Tertiary	583 (27.5)	420 (28.2)	1457 (28.9)	205 (29.1)	702 (28.9)	72 (25.2)	244 (27.9)	143 (28.8)	511 (29.4)
Missing	38 (1.8)	26 (1.7)	97 (1.9)	17 (2.4)	56 (2.3)	3 (1.0)	16 (1.8)	6 (1.2)	25 (1.4)
30-d Mortality	558 (26.3)	374 (25.2)	550 (10.9)	241 (34.2)	279 (11.5)	60 (21.0)	94 (10.7)	71 (14.3)	183 (10.5)

^a^ Unless otherwise indicated, data are expressed as No. (%) of patients.

^b^ Includes patients with their first recorded SARS-CoV-2 infection.

^c^ Based on *International Statistical Classification of Diseases and Related Health Problems, Tenth Revision* (*ICD-10*) codes from within 30 days to 3 years before start of study. See list of *ICD-10* codes for each comorbidity category in eTable 1 in Supplement 1.

**Figure 2.**
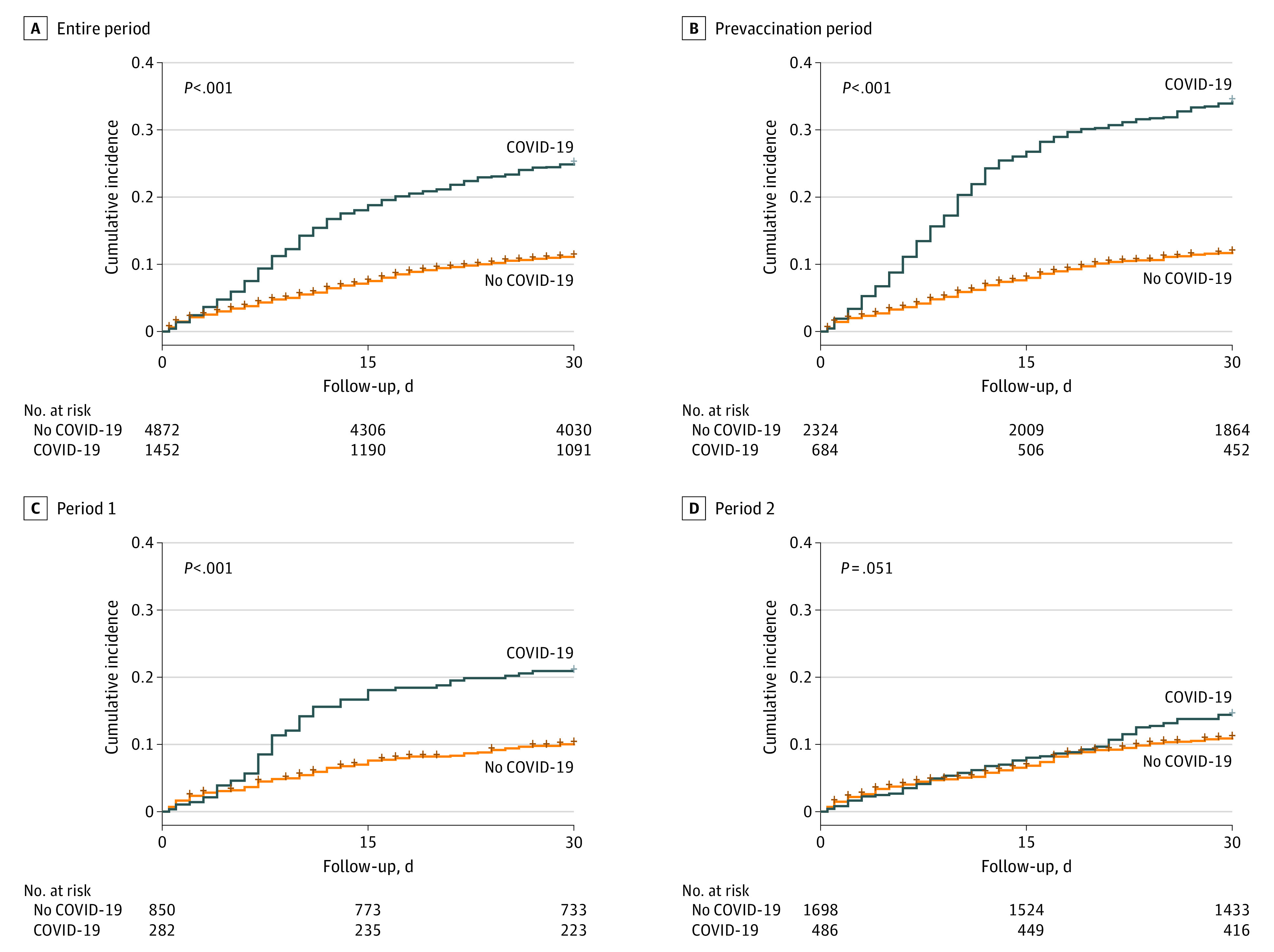
Unadjusted Kaplan-Meier Curves and Risk Tables for 30-Day All-Cause Mortality in the Matched Cohort The *P* value represents the result of significance testing performed using log-rank tests between the COVID-19 and non–COVID-19 groups.

**Figure 3.**
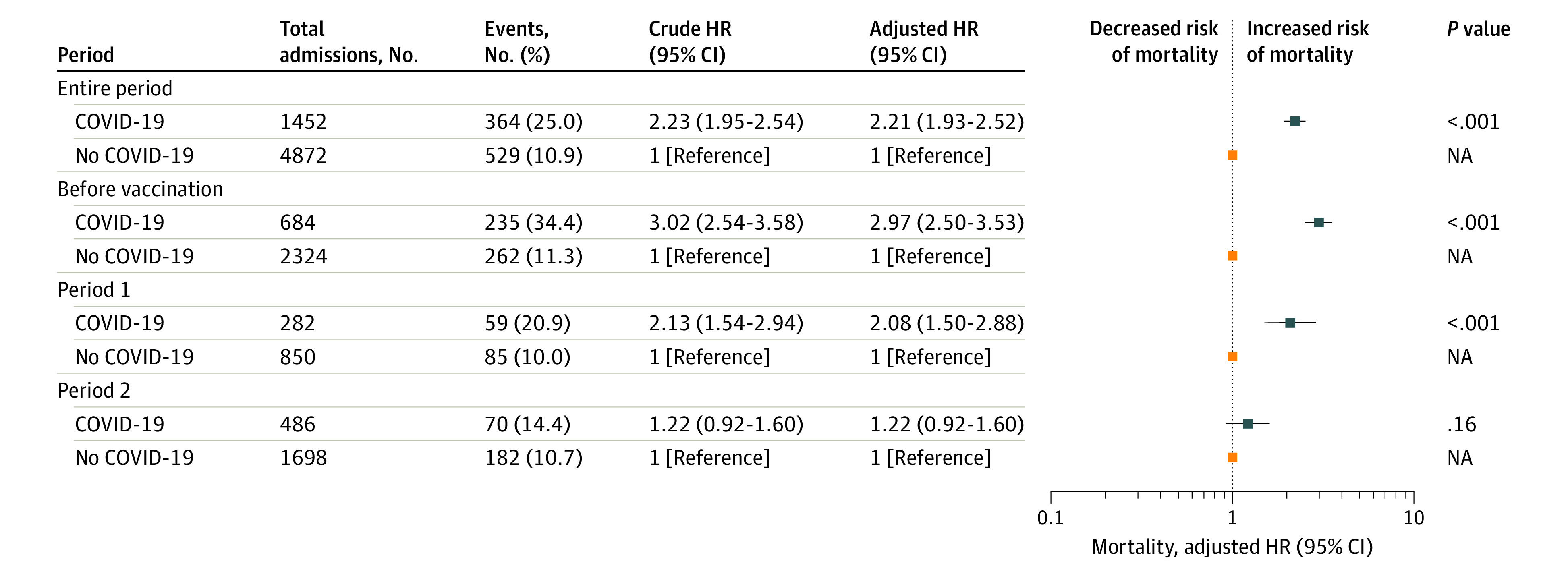
Forest Plot of Crude and Adjusted Hazard Ratios for 30-Day Mortality Comparing the COVID-19 and Non–COVID-19 Groups The Cox proportional hazards regression model was adjusted for age, sex, comorbidities, and educational level. Comorbidities included cancer, cardiovascular diseases, chronic kidney diseases, chronic lung diseases, diabetes, hypertension, and immunosuppression. Error bars indicate 95% CIs.

The analysis of mortality was restricted to 1452 COVID-19 and 4872 non–COVID-19 admissions with complete covariate information. The overall cumulative 30-day mortality was 25% (95% CI, 23%-27%) in the COVID-19 group compared with 11% (95% CI, 10%-12%) in the non–COVID-19 group (*P* < .001) (Figure 2A). In the prevaccination period, cumulative 30-day mortality was 34% (95% CI, 31%-38%) in the COVID-19 group compared with 12% (95% CI, 10%-13%) in the non–COVID-19 group (*P* < .001); and in period 1, 21% (95% CI, 16%-26%) compared with 10% (95% CI, 8%-12%), respectively (*P* < .001) (Figure 2B and C). In period 2, we observed no significant difference in 30-day mortality (14% [95% CI, 11%-17%] in the COVID-19 group and 11% [95% CI, 9%-13%] in the non–COVID-19 group; *P* = .05) (Figure 2D). The adjusted hazard ratio (AHR) for 30-day mortality decreased from 2.97 (95% CI, 2.50-3.53) during the prevaccination period to 2.08 (95% CI, 1.50-2.88) in period 1 and 1.22 (95% CI, 0.92-1.60) during period 2 (Figure 3). Except for chronic lung diseases (AHR, 1.39 [95% CI, 1.01-1.92]; *P* = .04), no significant interactions were detected between COVID-19 status and age (AHR, 1.00 [95% CI, 0.98-1.01]; *P* = .78), sex (AHR, 1.04 [95% CI, 0.80-1.36]; *P* = .76), and comorbid cancer (AHR, 1.02 [95% CI, 0.77-1.36]; *P* = .89), cardiovascular diseases (AHR, 1.00 [95% CI, 0.77-1.31]; *P* = .98), chronic kidney diseases (AHR, 1.28 [95% CI, 0.93-1.77]; *P* = .13), diabetes (AHR, 1.32 [95% CI, 0.98-1.77]; *P* = .07), hypertension (AHR, 1.22 [95% CI, 0.92-1.63]; *P* = .17), or immunosuppression (AHR, 1.16 [95% CI, 0.76-1.76]; *P* = .50) for mortality risk (eTable 4 in Supplement 1). The excess mortality in the COVID-19 group occurred during the first 30 days, with no significant increase in 31- to 90-day mortality observed between the 2 groups (eFigure 4 in Supplement 1). Sensitivity analyses showed similar results (eTable 5 in Supplement 1).

In the COVID-19 group, the percentage of 0 HFD was 31.7% (highest in prevaccination period at 43.5% and lowest at 15.8% in period 2) and 13.8% in the non–COVID-19 group (eFigure 5 in Supplement 1). Overall, the median HFD was 13 (95% CI, 12-15) days in the COVID-19 group and 23 (95% CI, 23-24) days in the non–COVID-19 group. In the COVID-19 group, median HFD was 6 (95% CI, 4-9) days in the prevaccination period, 13 (95% CI, 10-17) days in period 1, and 19 (95% CI, 17-21) days in period 2. For the non–COVID-19 group, HFD remained similar across all time periods (eFigure 5 in Supplement 1).

### Association Between SARS-CoV-2 Vaccination and 30-Day Mortality Among Nosocomial COVID-19 Admissions

The association between vaccination and 30-day mortality given a nosocomial infection of SARS-CoV-2 was analyzed in 1073 COVID-19 admissions during period 1 and 2, when vaccines were available (16 of 1089 admissions were excluded due to missing educational level data) (Table 2). Among these, patients with a primary course (≥2 doses) accounted for 789 (73.5%). The distribution of individuals with a primary course vs less than 2 doses was 235 vs 204 during period 1 and 554 vs 80 during period 2. Patients with a primary course of vaccination were older, mostly received the tozinameran vaccine, and had more comorbidities (Table 2).

**Table 2.** Demographic Characteristics and Outcome According to Vaccination Status Among All COVID-19 Admissions in Periods 1 and 2^a^

Characteristic	Vaccination status					
	Period 1		Period 2		Total	
	<2 Doses (n = 204)	≥2 Doses (n = 235)	<2 Doses (n = 80)	≥2 Doses (n = 554)	<2 Doses (n = 284)	≥2 Doses (n = 789)
Sex						
Women	101 (49.5)	124 (52.8)	46 (57.5)	270 (48.7)	147 (51.8)	394 (49.9)
Men	103 (50.5)	111 (47.2)	34 (42.5)	284 (51.3)	137 (48.2)	395 (50.1)
Age, median (IQR), y	75 (61-82)	78 (71-86)	75 (54-84)	82 (75-88)	75 (58-83)	81 (74-88)
No. of vaccine doses at admission date^b^						
0	156 (76.5)	1 (0.4)	65 (81.3)	0	221 (77.8)	1 (0.1)
1	46 (22.5)	2 (0.9)	15 (18.8)	0	61 (21.5)	2 (0.3)
2	2 (1.0)	167 (71.1)	0	91 (16.4)	2 (0.7)	258 (32.7)
3	0	65 (27.7)	0	293 (52.9)	0	358 (45.4)
4	0	0	0	168 (30.3)	0	168 (21.3)
5	0	0	0	2 (0.4)	0	2 (0.3)
Comorbidities^c^						
Cancer	36 (17.6)	42 (17.9)	5 (6.3)	125 (22.6)	41 (14.4)	167 (21.2)
Cardiovascular diseases	82 (40.2)	104 (44.3)	15 (18.8)	242 (43.7)	97 (34.2)	346 (43.9)
Chronic kidney diseases	26 (12.7)	31 (13.2)	4 (5.0)	85 (15.3)	30 (10.6)	116 (14.7)
Chronic lung diseases	28 (13.7)	33 (14.0)	7 (8.8)	92 (16.6)	35 (12.3)	125 (15.8)
Diabetes	40 (19.6)	52 (22.1)	8 (10.0)	133 (24.0)	48 (16.9)	185 (23.4)
Hypertension	108 (52.9)	152 (64.7)	30 (37.5)	357 (64.4)	138 (48.6)	509 (64.5)
Immunosuppression	8 (3.9)	14 (6.0)	2 (2.5)	20 (3.6)	10 (3.5)	34 (4.3)
Educational level						
Primary	47 (23.0)	65 (27.7)	25 (31.3)	136 (24.5)	72 (25.4)	201 (25.5)
Secondary	98 (48.0)	107 (45.5)	36 (45.0)	255 (46.0)	134 (47.2)	362 (45.9)
Tertiary	59 (28.9)	63 (26.8)	19 (23.8)	163 (29.4)	78 (27.5)	226 (28.6)
30-d Mortality	46 (22.5)	44 (18.7)	5 (6.3)	91 (16.4)	51 (18.0)	135 (17.1)

^a^ Excludes 16 of 1089 admissions due to missing information on educational level. Includes patients with their first recorded SARS-CoV-2 infection. Unless otherwise indicated, data are expressed as No. (%) of patients.

^b^ Distribution of COVID-19 vaccination types reported based on last received dose includes 673 patients had the tozinameran vaccine (Pfizer-BioNTech), 137 patients had had the elasomeran vaccine (Moderna), and 41 patients had had the ChAdOx1-S/nCoV-19 (recombinant) vaccine (AstraZeneca).

^c^ Based on *International Statistical Classification of Diseases and Related Health Problems, Tenth Revision* (*ICD-10*) codes from within 30 days to 3 years before start of study. See list of *ICD-10* codes for each comorbidity category in eTable 1 in Supplement 1.

The overall crude 30-day mortality was similar, including 51 of 284 patients (18.0%) with less than 2 doses, and 135 of 789 (17.1%) with the primary course. The AHR for 30-day mortality comparing the 2 groups was 0.64 (95% CI, 0.46-0.88). Although the absolute mortality was low among unvaccinated patients with COVID-19 during period 2, there was no strong evidence of vaccine effect modification on mortality between the periods (AHR, 2.54 [95% CI, 0.93-6.90]; *P* = .06).

## Discussion

This study showed that the excess mortality risk associated with nosocomial SARS-CoV-2 infection was substantial during the early phases of the pandemic; however, the effect was attenuated during the Omicron variant wave and after the introduction of vaccinations. In the prevaccination period, the incidence rate was 1.79 per 1000 patient-days, and the excess 30-day mortality was almost 3 times higher in the COVID-19 group compared with the non–COVID-19 group, resulting in around 20% absolute increased risk of death in patients acquiring a nosocomial SARS-CoV-2 infection. During period 1, the incidence rate was lowest at 0.94 per 1000 patient-days after introduction of vaccinations or strict IPC measures, but with 2 times higher relative excess risk of death and an absolute increased mortality risk of around 10%. In period 2, when the Omicron variant dominated transmission in a population with high immunization levels, no significantly higher risk of death was observed among hospitalized patients with SARS-CoV-2 infection, associated with the less severe Omicron variant and immunization.^5,16,17^ However, the incidence rate was highest at 2.25 per 1000 patient-days owing to the highly transmissible nature of the variant.^18^ Additionally, during the Omicron variant wave, patients with nosocomial SARS-CoV-2 infections still had lower HFD compared with their matched controls.

We compared the outcome of mortality among hospitalized patients with and without nosocomial SARS-CoV-2 infection and believe this to be a methodologically valid study design to assess excess mortality in hospitalized patients with nosocomial infections. Previously, most studies have compared mortality in individuals with nosocomial vs community-acquired infections; however, since hospitalized patients have a substantial risk of death irrespective of SARS-CoV-2 infection, such comparisons do not accurately assess the excess mortality.^2,3^ In our study, the 30-day mortality in the uninfected comparison cohort ranged from 10.3% to 11.4%, depending on the calendar period. The absolute mortality in patients with nosocomial SARS-CoV-2 in our study is in line with that of other studies that have analyzed mortality risk among nosocomial SARS-CoV-2–infected patients. In a systematic review of studies comparing community-acquired and nosocomial SARS-CoV-2 infections between January 2020 and February 2021,^2^ mortality was 37.8% among patients with nosocomial SARS-CoV-2. In a study from Germany, including 6652 patients with hospital-acquired SARS-CoV-2,^3^ odds of in-hospital mortality were 0.33 during the Omicron variant wave compared with the wildtype variant period.

In this study, the AHR for 30-day mortality was 0.66 (95% CI, 0.46-0.88) in patients with a confirmed nosocomial SARS-CoV-2 infection with at least 2 doses of SARS-CoV-2 vaccination compared with those with less than 2 doses, amounting to a protection of 40%. This is similar to a study of hospitalized patients with COVID-19 in England,^19^ which reported a mortality hazard of 0.56 (95% CI, 0.52-0.61) among patients with 2 vaccine doses compared with unvaccinated patients.

The proportion of SARS-CoV-2 infections in hospitalized patients was 7.6% in our study, compared with 11% to 20% reported from studies in the UK and Germany; the differences can be due to varying testing practices, definitions of nosocomial infections used, or implementation of IPC measures.^3,20,21^ The peak incidence of nosocomial infections following waves of high community transmission, with the highest peak observed in early 2022 due to spread of the Omicron variant, is in line with other reports.^3,22^ This has implications for timely implementation of IPC measures to mitigate the spread of SARS-CoV-2 in hospitals such as through use of personal protective equipment or improved testing practices.

### Strengths and Limitations

The strengths of this study lie in the use of complete data on positive SARS-CoV-2 test results, hospitalizations, comorbidity ascertainment, vaccination, sociodemographic characteristics, and mortality from all hospitals in Region Stockholm, increasing generalizability. Furthermore, the matching on age, sex, LOS in the hospital, hospital unit, and time of admission ensured that baseline characteristics and the risk of infection were similar in the cohorts. We adjusted for comorbidities and sociodemographic factors, which enhances the internal validity of our results. Similarity between groups for 31- to 90-day mortality, diagnosis distribution, and proportions of negative test results further favors comparability of the groups except for the exposure of SARS-CoV-2 infections.

There are also limitations to this study. Although we carefully matched patients and accounted for underlying diseases, unmeasured confounding is likely since we did not have access to detailed clinical status. As the circulating variant type and vaccination uptake in population changed over time, it was difficult to delineate the driver in reduction in mortality. Analyses were restricted (except for incidence rate estimates) to the first positive PCR test result for each patient in a mandatory reporting register to enhance internal validity of the results. However, we could not capture previous asymptomatic or untested SARS-CoV-2 infections or infections confirmed by home antigen tests. A negative SARS-CoV-2 test result on admission was confirmed for only half the cohort, which makes it possible that some patients classified as having nosocomial COVID-19 had community-onset infections. Information on symptomatic status of patients was lacking, leading to probable ascertainment bias, as reason for testing, whether due to symptoms or part of screening procedures, could not be determined. Estimates could also be influenced by varying treatment and personal protective equipment protocols across the study period and between hospitals. The case definition of nosocomial infection relied on the European Centre for Disease Prevention and Control definition, hence nosocomial infections with short incubation time or community-acquired infections with long incubation time could be misclassified.^13^ However, the sensitivity analysis using a 5-day cutoff showed consistent results. Finally, due to the low number of study participants, we could assess neither the association of nosocomial infections with mortality in vulnerable groups (eg, the immunosuppressed) nor the association between booster doses and patient outcomes.

## Conclusions

Implementation of IPC measures to mitigate excess mortality risk from nosocomial transmission should have a strong focus in any future setting of viral transmission when population immunity is low. In addition, implementation practices should be informed by assessing other factors associated with nosocomial infections such as longer LOS and secondary transmission risk. During the Omicron variant wave, after an extensive vaccination campaign, nosocomial infections were not independently associated with excess mortality, suggesting that the role of IPC measures may help prevent excess deaths with successful vaccination and/or less severe virus variants.

## Notes

Supplement 1. **eMethods.** Description of Matching Methodology **eTable 1.** *ICD-10* Codes for Comorbidities **eTable 2.** Proportion of Study Participants With a PCR Test Result for SARS-CoV-2 That Was Negative at Admission in the Matched Cohort **eTable 3.** Demographic Characteristics Between Nosocomial COVID-19 Admissions Excluded From Matched Cohort vs Included in Matched Cohort **eTable 4.** Interaction Terms for Age, Sex, Comorbidities, and Vaccine Effect Analyzed Using Cox Proportional Hazards Regression Models **eTable 5.** Sensitivity Analyses Using Cox Proportional Hazards Regression Models When Changing Nosocomial Definition With 5 Days Cutoff (Analysis 2) and Restriction of COVID-19 Admissions With PCR Cycle Threshold Values <30 (Analysis 3) **eFigure 1.** Incidence Rate per 1000 Population at Risk and Daily Count of Community SARS-CoV-2 Infections Between March 2020 Until September 2022 **eFigure 2.** Nosocomial SARS-CoV-2 Infections Among All Hospitalized Admissions With Any SARS-CoV-2 Infection **eFigure 3.** Distribution of ICD-10 Diagnosis Codes Between COVID-19 and Non–COVID-19 Group From the Matched Cohort **eFigure 4.** Unadjusted Kaplan Meier Curves for 31- to 90-Day Mortality in the Matched Cohort **eFigure 5.** Histogram Showing Percentage of Hospital-Free Days Among Matched Cohort for Whole Period and Stratified by Time Periods

Supplement 2.
Data Sharing Statement
