# Supplementary material for: Nosocomial SARS-CoV-2 Infections and Mortality During Unique COVID-19 Epidemic Waves
Source: JAMA Netw Open. 2023 Nov 10;6(11):e2341936. doi: 10.1001/jamanetworkopen.2023.41936 (PMC10638644; doi:10.1001/jamanetworkopen.2023.41936)
Supplement: Supplement 1. — eMethods. Description of Matching Methodology eTable 1. ICD-10 Codes for Comorbidities eTable 2. Proportion of Study Participants With a PCR Test Result for SARS-CoV-2 That Was Negative at Admission in the Matched Cohort eTable 3. Demographic Characteristics Between Nosocomial COVID-19 Admissions Excluded From Matched Cohort vs Included in Matched Cohort eTable 4. Interaction Terms for Age, Sex, Comorbidities, and Vaccine Effect Analyzed Using Cox Proportional Hazards Regression Models eTable 5. Sensitivity Analyses Using Cox Proportional Hazards Regression Models When Changing Nosocomial Definition With 5 Days Cutoff (Analysis 2) and Restriction of COVID-19 Admissions With PCR Cycle Threshold Values <30 (Analysis 3) eFigure 1. Incidence Rate per 1000 Population at Risk and Daily Count of Community SARS-CoV-2 Infections Between March 2020 Until September 2022 eFigure 2. Nosocomial SARS-CoV-2 Infections Among All Hospitalized Admissions With Any SARS-CoV-2 Infection eFigure 3. Distribution of ICD-10 Diagnosis Codes Between COVID-19 and Non–COVID-19 Group From the Matched Cohort eFigure 4. Unadjusted Kaplan Meier Curves for 31- to 90-Day Mortality in the Matched Cohort eFigure 5. Histogram Showing Percentage of Hospital-Free Days Among Matched Cohort for Whole Period and Stratified by Time Periods [file jamanetwopen-e2341936-s001.pdf]

Supplementary Online Content

Dave N, Sjöholm D, Hedberg P, et al. Nosocomial SARS-CoV-2 infections and mortality during unique COVID-19 epidemic waves. *JAMA Netw Open*. 2023;6(11):e2341936. doi:10.1001/jamanetworkopen.2023.41936

eMethods. Description of Matching Methodology
eTable 1. *ICD-10* Codes for Comorbidities
Table 2. Proportion of Study Participants With a PCR Test Result for SARS-COV-2 That Was Negative at Admission in the Matched Cohort
eTable 3. Demographic Characteristics Between Nosocomial COVID-19 Admissions Excluded From Matched Cohort vs Included in Matched Cohort
eTable 4. Interaction Terms for Age, Sex, Comorbidities, and Vaccine Effect Analyzed Using Cox Proportional Hazards Regression Models
eTable 5. Sensitivity Analyses Using Cox Proportional Hazards Regression Models When Changing Nosocomial Definition With 5 Days Cutoff (Analysis 2) and Restriction of COVID-19 Admissions With PCR Cycle Threshold Values <30 (Analysis 3)
eFigure 1. Incidence Rate per 1000 Population at Risk and Daily Count of Community SARS-CoV-2 Infections Between March 2020 Until September 2022
eFigure 2. Nosocomial SARS-CoV-2 Infections Among All Hospitalized Admissions With Any SARS-CoV-2 Infection
eFigure 3. Distribution of ICD-10 Diagnosis Codes Between COVID-19 and Non–COVID-19 Group From the Matched Cohort
eFigure 4. Unadjusted Kaplan Meier Curves for 31- to 90-Day Mortality in the Matched Cohort
eFigure 5. Histogram Showing Percentage of Hospital-Free Days Among Matched Cohort for Whole Period and Stratified by Time Periods

This supplementary material has been provided by the authors to give readers additional information about their work.

© 2023 Dave N et al. *JAMA Network Open*.

eMethods. Description of Matching Methodology

A nosocomial infection was defined as a patient testing PCR positive for SARS-CoV-2 at least 8 days from admission or within 2 days after discharge given a length of stay of at least 8 days. All admissions where a patient did not test PCR positive for SARS-CoV-2 were considered as non-COVID-19 admissions. Upon a positive PCR for SARS-CoV-2, that admission was considered a COVID-19 admission from the date of positive PCR test.

For the COVID-19 admissions, the index date was the date of the positive SARS-CoV-2 PCR test. For the non-COVID-19 admissions, the index date was the date of admission plus days it took for the matched COVID-19 admission to test positive. To create the cohort, each COVID-19 admission was matched to up to 5 non-COVID-19 admissions on:

- age (± 2 years),
- sex (exact matching),
- calendar time of admission (non-COVID-19 admission date within ±30 days of matched COVID-19 admission date, as well as within the same time period),
- days from admission until index date,
- patients from both non-COVID-19 and COVID-19 admissions to be in the same clinic on the index date.

Below we present a matching scenario, whereby Patient A tested positive for SARS-CoV-2 on day 35 after admission, fulfilling the definition of nosocomial SARS-CoV-2 infection and was marked as the COVID-19 admission. Patients B-E represent different matching scenarios. Patient B was discharged before 35 days of stay and was thus not eligible as a matched non-COVID-19 admission. Patient E tested positive, at which point will be censored as a non-COVID-19 admission to Patient A and regarded as a COVID-19 admission.

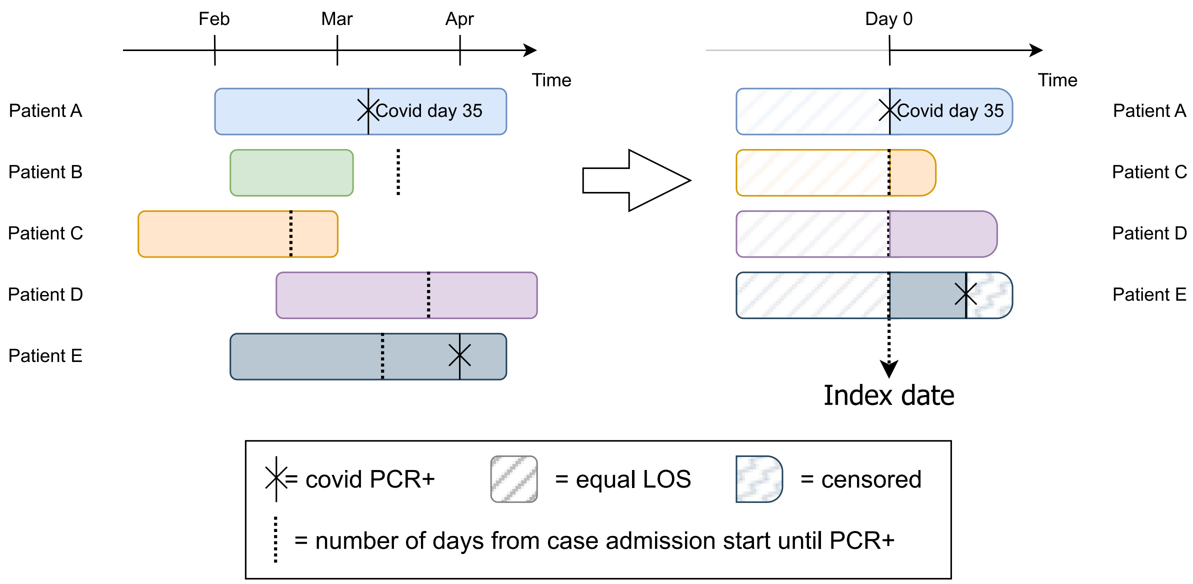

© 2023 Dave N et al. *JAMA Network Open*.

eTable 1. *ICD-10* Codes for Comorbidities

**Comorbidity category**	**ICD-10 codes[a]**
Cancer	C0-C1, C4-C7, C20-C26, C30-C34, C37-C39, C80-C96
Chronic kidney disease	N18
Chronic lung diseases	I26, I27.0, I27.2, J47, J70.2-J70.4, J84, J98.2, J99.0, M05.1
Cardiac diseases	I05-I08, I20-I22, I24-I28, I34-I37, I42, I44-I50
Diabetes	E10-E14
Hypertension	I10-I15
Immunosuppression	D70-D72, D73.0, D80-D84, Z51.0, Z51.1

[a] Comorbidities included in the study are based on ICD-10 codes recorded from 3 years before the start of study.

© 2023 Dave N et al. *JAMA Network Open*.

eTable 2. Proportion of Study Participants With a PCR Test Result for SARS-COV-2 That Was Negative at Admission in the Matched Cohort[a]

Included in matched cohort analysis								
	**Whole Period**		**Pre-Vaccination Period**		**Period 1**		**Period 2**	
	**COVID-19 (N=1487)**	**Non-COVID-19 (N=5044)**	**COVID-19 (N=705)**	**Non-COVID-19 (N=2427)**	**COVID-19 (N=286)**	**Non-COVID-19 (N=876)**	**COVID-19 (N=496)**	**Non-COVID-19 (N=1741)**
**Negative PCR test**	792 (53.3)	2760 (54.7)	430 (61.0)	1428 (58.8)	140 (49.0)	488 (55.7)	222 (44.8)	844 (48.5)

[a] Testing at admission was defined as testing negative with PCR for SARS-CoV-2 +/- days within admission. Data is based on information from the Quality Register for SARS-CoV-2 (Covid-19) that collects data from laboratories on analyses for SARS-CoV-2 tests performed in Stockholm County.

© 2023 Dave N et al. *JAMA Network Open*.

eTable 3. Demographic Characteristics Between Nosocomial COVID-19 Admissions Excluded From Matched Cohort vs Included in Matched Cohort

	**Excluded (N=633)**	**Included (N=1487)**	**Total (N=2120)**
**Sex, No. (%)**			
Female	289 (45.7%)	783 (52.7%)	1072 (50.6%)
Male	344 (54.3%)	704 (47.3%)	1048 (49.4%)
**Age, median (IQR), y**			
Median [IQR]	74 [60, 84]	81 [75, 87]	80 [71, 87]
**Number of vaccine doses at admission date, No. (%)**			
0	402 (63.5%)	856 (57.6%)	1258 (59.3%)
1	18 (2.8%)	46 (3.1%)	64 (3.0%)
2	99 (15.6%)	164 (11.0%)	263 (12.4%)
3	77 (12.2%)	287 (19.3%)	364 (17.2%)
4	37 (5.8%)	132 (8.9%)	169 (8.0%)
5	0 (0%)	2 (0.1%)	2 (0.1%)
**Comorbidities, No. (%)**			
Cancer	140 (22.1%)	322 (21.7%)	462 (21.8%)
Cardiovascular diseases	249 (39.3%)	697 (46.9%)	946 (44.6%)
Chronic kidney diseases	93 (14.7%)	244 (16.4%)	337 (15.9%)
Chronic lung diseases	104 (16.4%)	288 (19.4%)	392 (18.5%)
Diabetes	144 (22.7%)	368 (24.7%)	512 (24.2%)
Hypertension	342 (54.0%)	947 (63.7%)	1289 (60.8%)
Immunosuppression	44 (7.0%)	86 (5.8%)	130 (6.1%)
**Education level, No. (%)**			
Primary	154 (24.3%)	413 (27.8%)	567 (26.7%)
Secondary	304 (48.0%)	628 (42.2%)	932 (44.0%)
Tertiary	163 (25.8%)	420 (28.2%)	583 (27.5%)
Missing	12 (1.9%)	26 (1.7%)	38 (1.8%)
**Mortality, No. (%)**			
30 day	184 (29.1%)	374 (25.2%)	558 (26.3%)

Abbreviation: IQR = Interquartile range

© 2023 Dave N et al. *JAMA Network Open*.

eTable 4. Interaction Terms for Age, Sex, Comorbidities, and Vaccine Effect Analyzed Using Cox Proportional Hazards Regression Models[a]

	**Adjusted HR (95% CI)**
**Interaction with age and COVID-19 status**	1.00 (0.98 – 1.01)
**Interaction with sex and COVID-19 status**	1.04 (0.80 – 1.36)
**Interaction with comorbid cancer and COVID-19 status**	1.02 (0.77 – 1.36)
**Interaction with comorbid cardiovascular diseases and COVID-19**	1.00 (0.77 – 1.31)
**Interaction with comorbid chronic kidney diseases and COVID-19 status**	1.28 (0.93 – 1.77)
**Interaction with comorbid chronic lung diseases and COVID-19 status**	1.39 (1.01 – 1.92)
**Interaction with comorbid diabetes and COVID-19 status**	1.32 (0.98 – 1.77)
**Interaction with comorbid hypertension and COVID-19 status**	1.22 (0.92 – 1.63)
**Interaction with comorbid immunosuppression and COVID-19 status**	1.16 (0.76 – 1.76)
**Time adjusted vaccine effect model[b]**	0.79 (0.55 – 1.13)
**Interaction with vaccine status and time period[b]**	2.54 (0.93 – 6.90)

[a] Cox regression model adjusted for age, sex, comorbidities, and education level.

[a] Cox regression model adjusted for age, sex, time period, comorbidities, and education level.

Abbreviation: CI = Confidence Interval, HR = Hazard Ratio

© 2023 Dave N et al. *JAMA Network Open*.

eTable 5. Sensitivity Analyses Using Cox Proportional Hazards Regression Models When Changing Nosocomial Definition With 5 Days Cutoff (Analysis 2) and Restriction of COVID-19 Admissions With PCR Cycle Threshold Values <30 (Analysis 3)[a]

Period	Analysis 1: Original analysis				Analysis 2: Nosocomial definition with 5 days cut off[b]				Analysis 3: COVID-19 admissions with ct values ≤ 30 + matched non-COVID-19 admissions[c]			
	CRUDE HR (95% CI)		ADJUSTED HR (95% CI)		CRUDE HR (95% CI)		ADJUSTED HR (95% CI)		CRUDE HR (95% CI)		ADJUSTED HR (95% CI)	
	COVID-19	Non-COVID-19	COVID-19	Non-COVID-19	COVID-19	Non-COVID-19	COVID-19	Non-COVID-19	COVID-19	Non-COVID-19	COVID-19	Non-COVID-19
**Whole Period**	2.23 (1.95 to 2.54)	Reference	2.21 (1.93 to 2.52)	Reference	2.33 (2.08 to 2.60)	Reference	2.33 (2.09 to 2.60)	Reference				
**Pre-vaccination**	3.02 (2.54 to 3.58)	Reference	2.97 (2.50 to 3.53)	Reference	3.31 (2.85 to 3.83)	Reference	3.29 (2.84 to 3.81)	Reference				
**Period 1**	2.13 (1.54 to 2.94)	Reference	2.08 (1.50 to 2.88)	Reference	2.37 (1.79 to 3.14)	Reference	2.37 (1.79 to 3.14)	Reference				
**Period 2**	1.22 (0.92 to 1.60)	Reference	1.22 (0.92 to 1.60)	Reference	1.19 (0.95 to 1.50)	Reference	1.19 (0.95 to 1.50)	Reference	1.21 (0.83 to 1.76)	Reference	1.18 (0.81 to 1.72)	Reference

[a] Cox regression model adjusted for age, sex, comorbidities, and education level. Comorbidities included: cancer, chronic kidney diseases, chronic lung diseases, diabetes, cardiovascular diseases, hypertension, and immunosuppression.

[b] Nosocomial SARS-CoV-2 infection defined as a positive SARS-CoV-2 PCR test ≥5 days after admission or up to 2 days after discharge given a length of stay in hospital of at least 5 days. 2149 COVID-19 admissions and 7814 matched non-COVID-19 admissions were identified, out of which, we excluded 323 with missing education level data. The final analysis included 2100 COVID-19 admissions and 7540 matched non-COVID-19 admissions.

[b] We identified all cases with PCR sample ct value below 30 and when ct value data was unavailable, sequencing data was used, as obtaining a sequencing result indicates sufficient viral load for detection. Out of 1487 COVID-19 admissions, 221 COVID-19 admissions with ct value ≤30 and 800 matched non-COVID-19 admissions identified in Period 2. Analysis restricted to Period 2 due to incomplete ct value data for other time-periods. The final analysis included 214 COVID-19 admissions and 778 matched non-COVID-19 admissions, excluding 29 admissions with missing education level data.

Abbreviation: CI = Confidence Interval, CT = Cycle Threshold, HR = Hazard Ratio

© 2023 Dave N et al. *JAMA Network Open*.

eFigure 1. Incidence Rate per 1000 Population at Risk and Daily Count of Community SARS-CoV-2 Infections Between March 2020 Until September 2022[a]

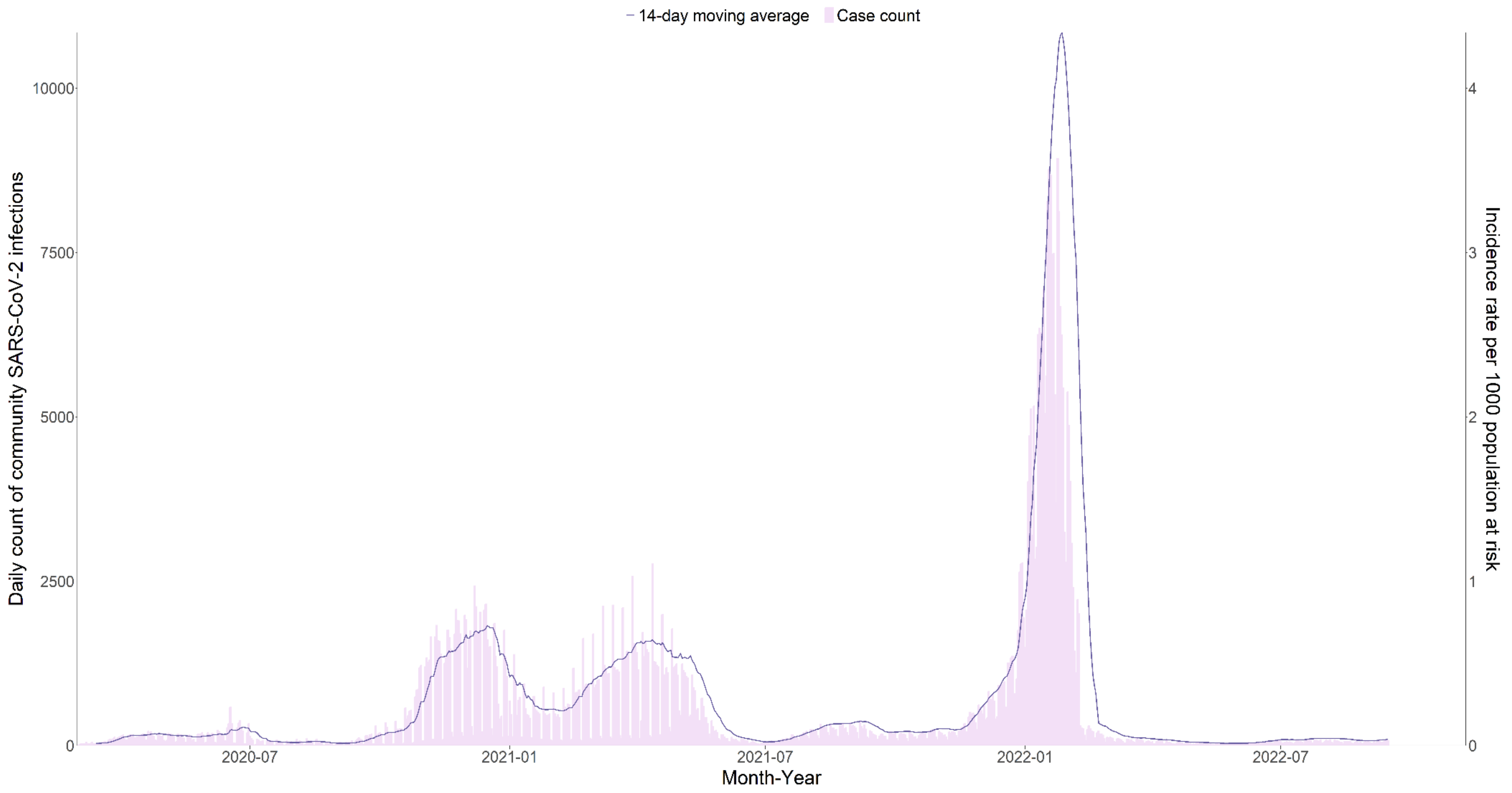

[a] All first positive PCR results among community SARS-CoV-2 infections

© 2023 Dave N et al. *JAMA Network Open*.

eFigure 2. Nosocomial SARS-CoV-2 Infections Among All Hospitalized Admissions With Any SARS-CoV-2 Infection[a]

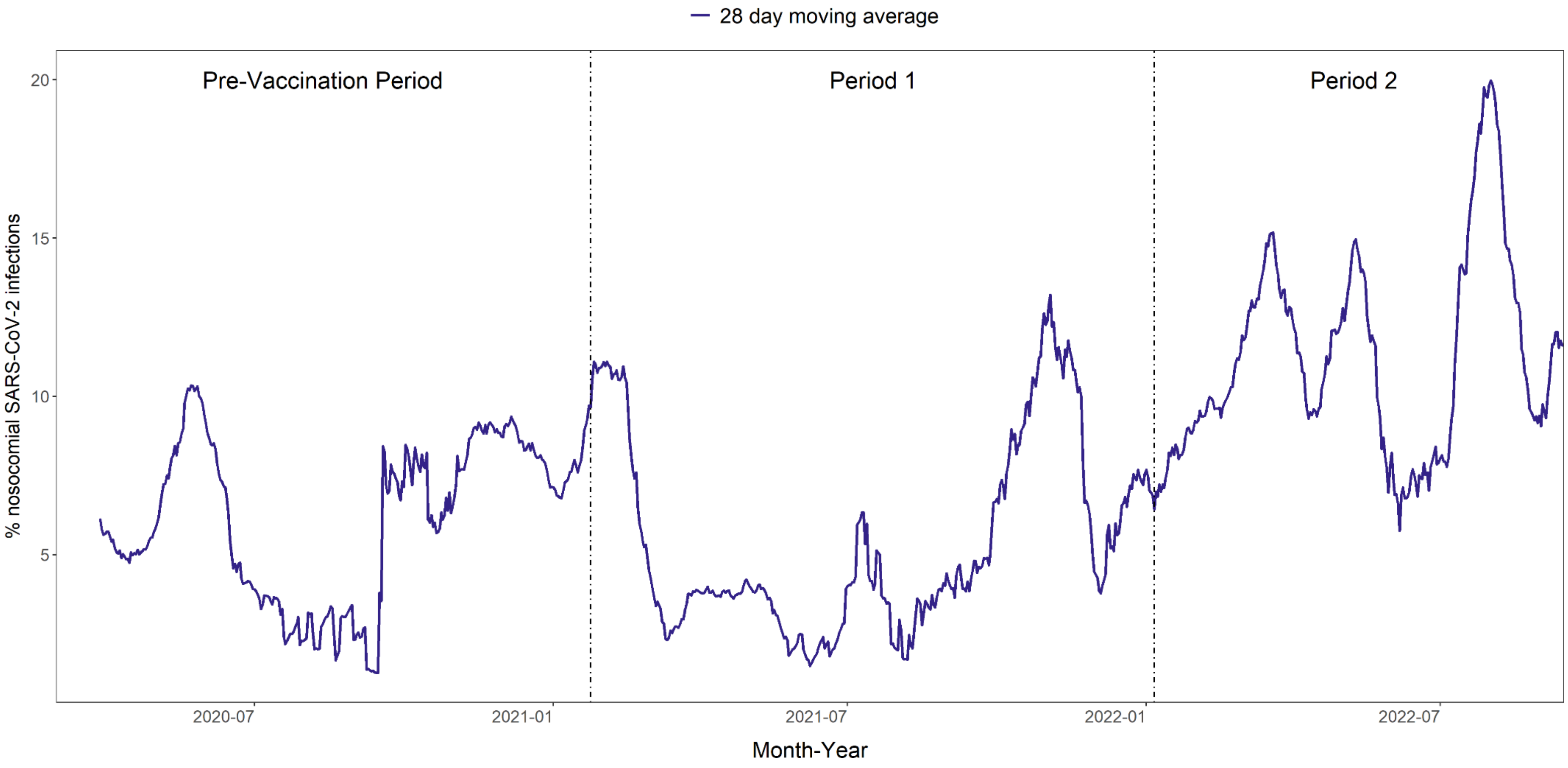

[a] Proportion of community-acquired, indeterminate, or nosocomial SARS-CoV-2 infection (any SARS-CoV-2 infection) among all hospitalized patients

© 2023 Dave N et al. *JAMA Network Open*.

eFigure 3. Distribution of ICD-10 Diagnosis Codes Between COVID-19 and Non–COVID-19 Group From the Matched Cohort

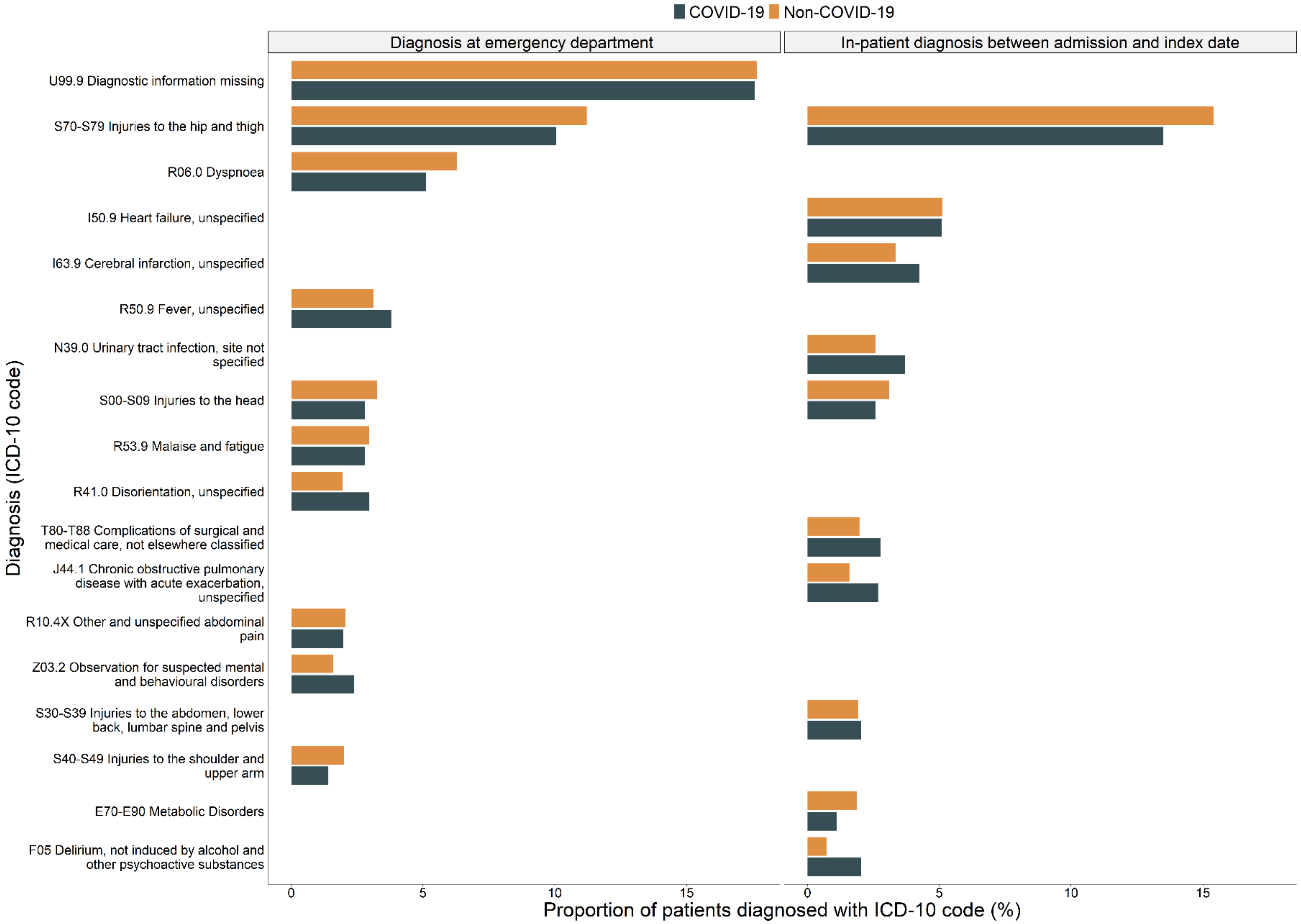

© 2023 Dave N et al. *JAMA Network Open*.

eFigure 4. Unadjusted Kaplan Meier Curves for 31- to 90-Day Mortality in the Matched Cohort

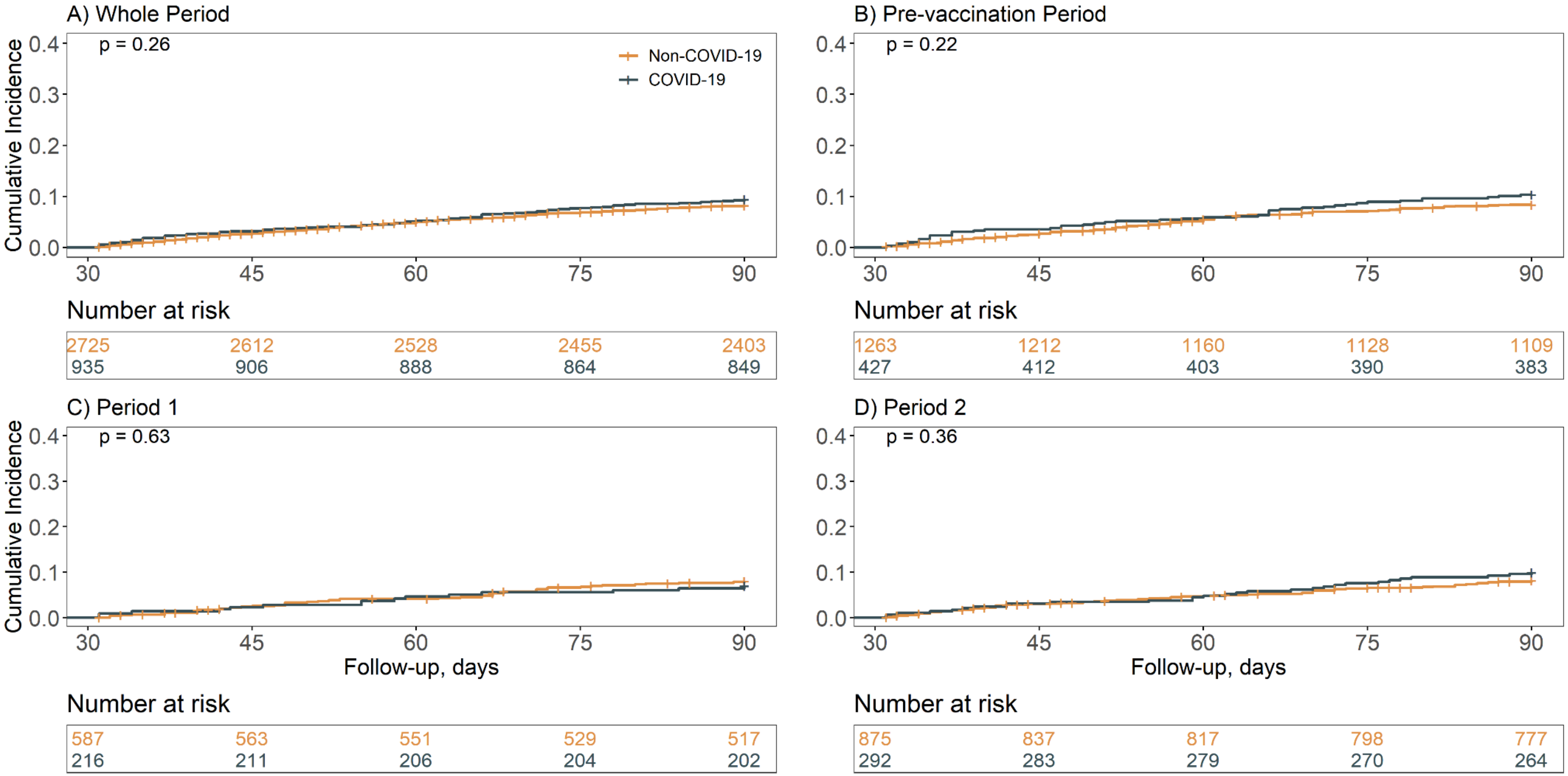

A. Unadjusted Kaplan Meier curves and risk tables for 31-90-day mortality for the whole study period. **B.** Unadjusted Kaplan Meier curves and risk tables for 31-90-day mortality for pre-vaccination period. **C.** Unadjusted Kaplan Meier curves and risk tables for 31-90-day mortality for period 1. **D.** Unadjusted Kaplan Meier curves and risk tables for 31-90-day mortality for period 2. The P-value represents the result of significance testing performed using log-rank tests between the COVID-19 and non-COVID-19 group.

© 2023 Dave N et al. *JAMA Network Open*.

eFigure 5. Histogram Showing Percentage of Hospital-Free Days Among Matched Cohort for Whole Period and Stratified by Time Periods

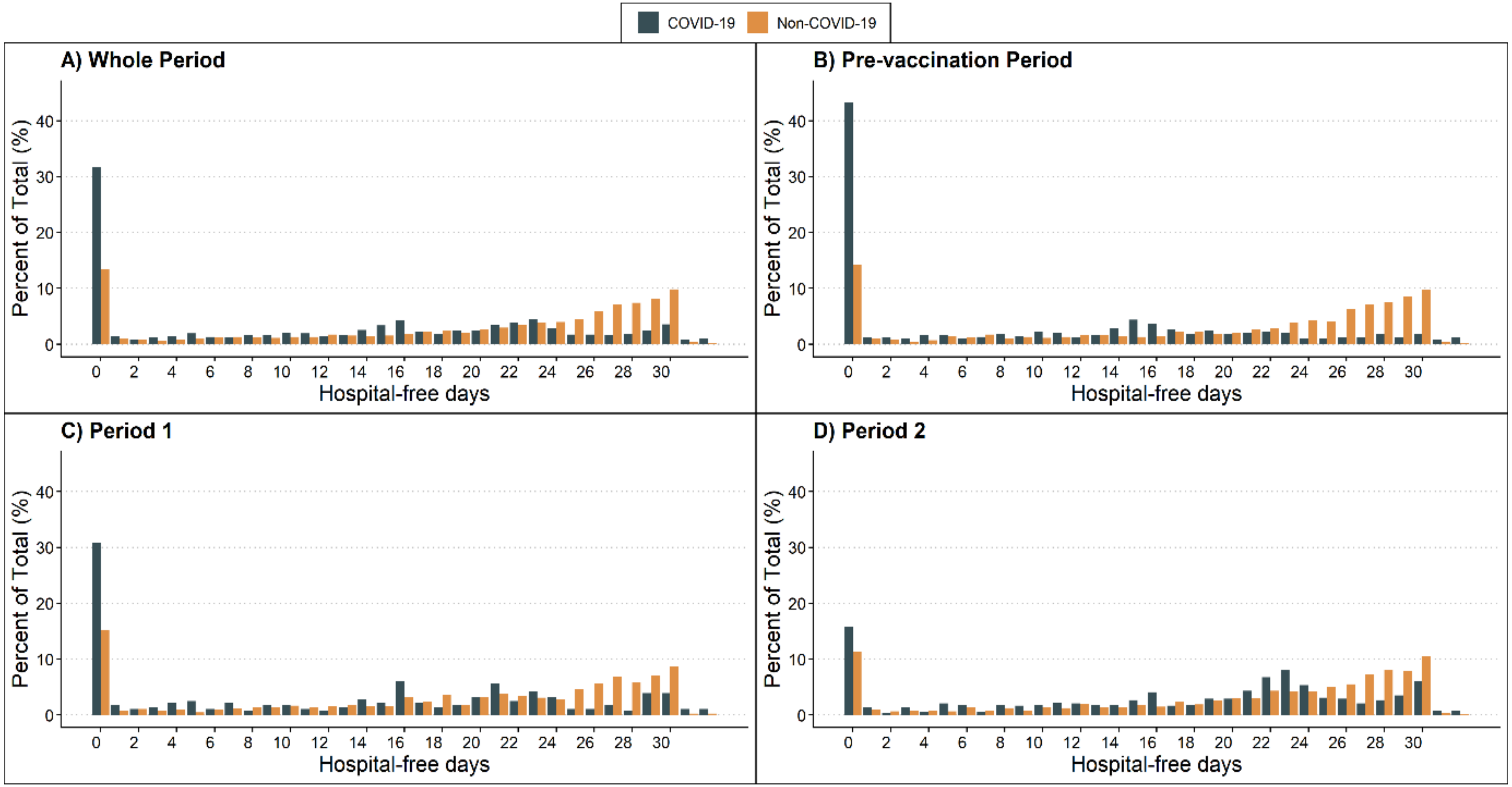

© 2023 Dave N et al. *JAMA Network Open*.
