# Supplementary material for: Nosocomial SARS-CoV-2 Infections and Mortality During Unique COVID-19 Epidemic Waves
Source: JAMA Netw Open. 2023 Nov 10;6(11):e2341936. doi: 10.1001/jamanetworkopen.2023.41936 (PMC10638644; doi:10.1001/jamanetworkopen.2023.41936)
Supplement: Supplement 2. — Data Sharing Statement [file jamanetwopen-e2341936-s002.pdf]

Data Sharing Statement

Dave. Nosocomial SARS-CoV-2 Infections and Mortality During Unique COVID-19 Epidemic Waves. *JAMA Netw Open*. Published November 10, 2023.
doi:10.1001/jamanetworkopen.2023.41936

Data
Data available: No

Additional Information
Explanation for why data not available: The data on individual participants used in this study were obtained using ethical approval and therefore it cannot be shared freely or publicly.
